# A Simple Measure of Human Development: The Human Life Indicator

**DOI:** 10.1111/padr.12205

**Published:** 2018-11-06

**Authors:** Simone Ghislandi, Warren C. Sanderson, Sergei Scherbov

for much of the twentieth century, statistical measurements of human development emphasized economic magnitudes. In 1990, the United Nations offered an alternative and more comprehensive way of measuring human development, the Human Development Index (HDI) (UNDP 1990). The motivation behind the production of the HDI was that economic magnitudes alone provided too narrow a basis for assessing human development. The HDI represents a compromise between comprehensiveness and measurability. In the HDI, the level of human development is conceptualized as having three components: health, education, and economic conditions. These are quantified at the country level using four indicators: life expectancy at birth, mean and expected years of schooling, and the logarithm of Gross National Income per capita (PPP$).^i^ The mean and expected years of schooling are combined into a single education index and it is this aggregate that enters the computation of the HDI. These indicators are then rescaled by using “goalposts” (i.e., the rescaled value is calculated as the observed value of each indicator minus the lower goalpost, all divided by the difference between the upper and lower goalposts). The goalposts often change with the HDI version. Although the details of how the HDI is computed have changed from time to time (see Appendix Table A1 for details), the UN has been publishing the “Human Development Reports” regularly since 1990, providing the values of the HDIs for approximately 180 countries around the world and ranking them accordingly (UNDP 1990, 2005, 2010, 2011, 2013, 2014, 2016b).

## Selected problems with the HDI

The 1990 Human Development Report was indeed a seminal publication and numerous articles on measuring human development have since been published. Despite its success, the methodology of the HDI has been widely criticized (see, for example, Kelley 1991; McGillivray 1991; Dasgupta and Weale 1992; Castles 1998; Sagar and Najam 1998; Booysen 2002; Lutz and Goujon 2004; Kovacevic 2011; Wolff, Chong, and Auffhammer 2011). This is not surprising since its construction involves a series of assumptions regarding weighting, functional forms, and the selection of the policy components. In some cases, criticisms and debates have helped improve the index. Nevertheless, some limitations of the HDI remain. We focus on four of them here: (1) measurement errors in its components, with the economic component having the greatest measurement error, (2) historical inconsistency, (3) unjustified trade‐offs across its components, and (4) the correlation of its components.

## Errors in the components

One important limitation of the HDI arises because of the high level of measurement error in each one of its components (see, for example, Castles 1998). Wolff, Chong, and Auffhammer (2011) conclude that when ranking countries by HDI, 34 percent of them are misclassified due to data errors. Importantly, in their analysis of the economic component, GNI per capita (PPP$) has the highest measurement error, while life expectancy is the most precise among the three components. It is not surprising that GNI per capita has the highest measurement error. Because of technical considerations, GNI per capita must be calculated for all countries at the same time. Because the collection of comparable prices for nearly 200 countries is costly and the methodology of combining all those data is complex and based on many subjective assumptions, the computation of GNI per capita is done only periodically.

In particular, the last round of GNI per capita (PPP$) computation was based on 2011 prices and was released in 2014. The previous round was based on 2005 prices. Between benchmark years GNI per capita is extrapolated. When the 2011 data were released it was found that the data extrapolated from the 2005 benchmark differed significantly from what was observed based on the 2011 benchmark. Deaton and Aten (2017) argue that some of that difference was due to methodological errors in the 2005 benchmark data. Any random variability or methodological errors in the benchmark data then are propagated through extrapolation until the next benchmarks are released. GNI per capita based on the 2011 methodology assessed incomes across the world's countries as being considerably more equal than when those figures were computed based on the 2005 methodology.

## Structural changes in the goalpost values and the functional form of the index

During the past decade, the details of how the HDI is computed have frequently changed.

These changes ultimately reflect the lack of consensus on the relative importance of the factors influencing human development. For example, it is not clear whether in calculating human development, the geometric or arithmetic average of the mean and expected years of schooling should be used. In assessing human development, there is no benefit for having a GNI per capita above $75,000 (the actual goalpost for income in the 2016 HDI). Instead of $75,000 could that income level be $85,000, or $65,000? This lack of consensus suggests the possibility that the methodology of the HDI will continue to be contested and that changes will continue to be made.

A historical series of HDIs can be produced holding the functional form of the HDI fixed as well as the goalpost values. However, different historical series result each time the functional form of the HDI or the goalposts are changed. Historical series of HDI values depend on historical series of GNI per capita, and it is not clear how well those data reflect the growth of real GNI per capita using local currency prices. A promising approach to long‐run GNI measurement has recently been suggested by Bolt and colleagues (2018).

## Unexplained trade‐offs in HDI indices

**Figure 1:**
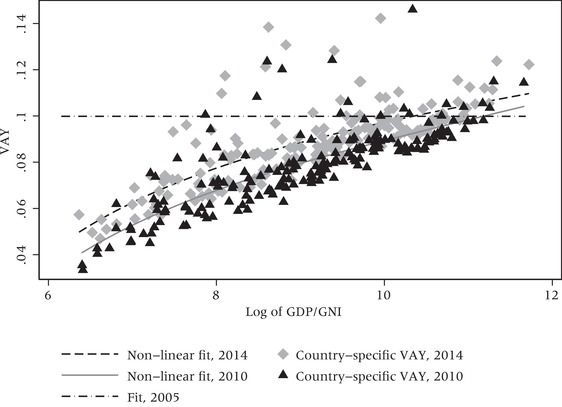
Value of one additional year of life as percent of GNI per capita (VAY), as implied by UNDP (2005, 2010, 2014) NOTES: Value of one additional year of life as a percent of income per capita (VAY). The value of one additional year of life is the reduction in per capita income required to keep the HDI constant when life expectancy at birth is increased by one year. All HDI countries. HDI formulas used in 2005, 2010, and 2014. Horizontal axis: Log of GNI per capita. Lines show polynomial fits. For 2005, the line is horizontal since the VAY is constant at about 0.1. Data are from UNDP (2016a).

An important technical criticism relates to the implied trade‐offs across the HDI's components, such as the amount of GNI per capita needed to compensate for one year loss of life expectancy. The value of one life year gained in terms of GNI per capita varies across countries and years (according to the different formulas for the HDI). The magnitude of the trade‐offs depends crucially on the formula used to combine the different components, but the value given to one additional year of life in terms of GNI per capita is generally lower the poorer the country (Figure 1).

This has important policy consequences. Take for example Zimbabwe, the country with the lowest income in 2010. Ravallion (2012) shows that given the value of the HDI and its components, the country in 2010 would have obtained a higher HDI by implementing a policy that increased income by only 0.52 PPP$ per capita than one that increased life expectancy of one entire year. This implies that if a poor country decided to use the HDI to determine its stage of development, income would count much more than life length. For example, using the methodology of the 2016 Human Development Report, we examine the trade‐offs for four countries, two with relatively high HDI indices (Austria and Italy) and two with relatively low ones (Haiti and Senegal). Changes that would keep the value of the HDI constant include increasing life expectancy at birth in Austria by one year and decreasing Austria's GNI per capita by 9.3 percent. The decreases in GNI per capita that keep HDI constant when life expectancy at birth increases by one year are 8.6 percent for Italy, 6.3 percent for Senegal, and 6.2 percent for Haiti.

**Table 1:** Trade‐offs in the HDI, four countries

	Austria				Italy			
	(HDI = 0.893)				(HDI = 0.887)			
HDI Components	LE	MYS	EYS	GNI/POP	LE	MYS	EYS	GNI/POP
	81.6	11.3	15.9	43,609	83.3	10.9	16.3	33,573
Trade‐offs (HDI constant)								
Trade‐off 1		+ 1 yr		–21.2%		+ 1 yr		–20.4%
Trade‐off 2	–2.4 yr	+ 1 yr			–2.5 yr	+ 1 yr		
Trade‐off 3			+1 yr	–18.1%			+1 yr	–17.4%
Trade‐off 4	–2.0 yr		+1yr		–2.1 yr		+1yr	

	Senegal				Haiti			
	(HDI = 0.495)				(HDI = 0.493)			
HDI Components	LE	MYS	EYS	GNI/POP	LE	MYS	EYS	GNI/POP
	66.9	2.8	9.5	2,250	63.1	5.2	9.1	1,657
Trade‐offs (HDI constant)								
Trade‐off 1		+ 1 yr		–23.3%		+ 1 yr		–18.4%
Trade‐off 2	–4 yr	+ 1 yr			–3.1 yr	+ 1 yr		
Trade‐off 3			+1 yr	–20.1%			+1 yr	–15.8%
Trade‐off 4	–3.4 yr		+1yr		–2.6 yr		+1yr	

SOURCE: UNDP 2016b and authors’ calculations.

NOTES: HDI components and changes in them that keep HDI constant. HDI = Human Development Index. LE = Life Expectancy. MYS = Mean Years of Schooling; EYS = Expected Years of Schooling; GNI/POP = Per Capita GNI.

Table 1 shows other examples of these trade‐offs. The reductions in GNI per capita that keep the HDI constant when the mean years of schooling are increased by one year are large. In Austria, the reduction is 21.2 percent of GNI per capita. The decrease in GNI per capita in Italy is 20.4 percent, in Senegal 23.3 percent, and in Haiti 18.4 percent. In particular, Senegal had mean years of schooling of 2.8. Its value of the HDI would be the same if it increased it to 3.8 and reduced its GNI by almost one‐quarter. In Austria, a reduction in GNI per capita of 21.2 percent would be counterbalanced by an increase in the mean years of schooling of one year, from 11.3 to 12.3. Are all these country‐specific trade‐off values really meaningful?

## The redundancy of the underlying indices

A further relevant concern is related to the redundancy of HDI components. Life expectancy, GNI, and education are strongly correlated with one other, both empirically and conceptually (McGillivray 1991; Ogwang 1994; Sen 1998; Ogwang and Abdou 2003). This is not surprising. People who are more educated tend to be richer and, on average, experience longer life spans. This is known as the “socioeconomic gradient” and represents a very pervasive phenomenon in virtually all societies around the world (Marmot 2005). In itself, redundancy does not pose a problem for the reliability of the index. However, it does question the efficiency of the HDI, suggesting that the composite index might not reveal more than its single components. Similar results could then be achieved through a less information‐demanding approach.

## Measuring human life and its distribution: The Human Life Indicator

If one takes both history and measurability seriously, the best approach is to “keep it simple,” reducing the redundancies related to a multidimensional index and providing data that can be sufficiently stable as to allow comparisons across time. This is true also for the measurement of human development.

Life expectancy represents a good candidate for this purpose. Since it is one of the three components included in the HDI, this strategy would require dropping education and GNI per capita. This would be problematic only to the extent that relevant information is lost. However, the strong correlation among the components reduces significantly the related information loss.

**Figure B1:**
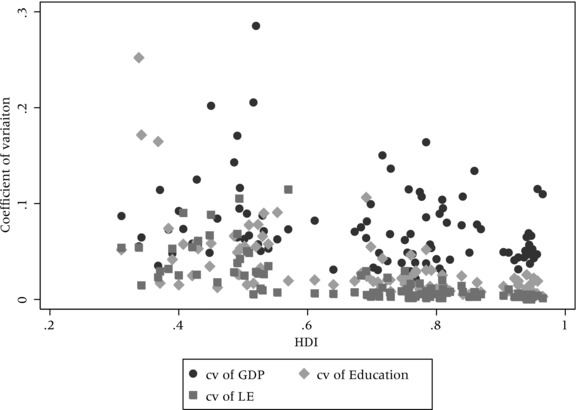
Coefficients of variation of measurement errors of Education, Life Expectancy, and Gross Domestic Product, over HDI NOTES: Coefficients of variation (CV) for an index for country i: σi/μi, where σ is the standard error of the country‐specific measurement error, and μ is the country‐specific mean. Countries are ordered according to their Human Development Index (HDI). Measurement errors are from Wolff, Chong, and Auffhammer (2011). The graph shows that measurement errors generally decrease for countries with higher HDI, and that life expectancy is associated with lower measurement errors, especially for high HDI countries.

The use of the sole health component of human development instead of a composite index helps to answer some of the more technical criticisms of the HDI defined above, since:

1. It does not depend on any trade‐off, implying that the economic and schooling components matter *only to the extent that* they can influence life conditions and mortality. Note that this does not imply that income and education are not important. Rather, an increase in the economic and educational levels of a country should be considered as relevant only when they translate into an improved life‐span for individuals. This view is consistent with the approach to development based on the idea that we should be seeing “incomes and commodities” not as something that “people have reason to value intrinsically,” but rather as “instruments,” i.e., as “means to other ends,” this end being a good and long life (Sen 1998). Sen explicitly suggests that mortality should be considered as an indicator of economic success and failure (Sen 1998).
2. Its measurement errors are smaller than the ones associated with education and income. The measurement errors for the three indicators of the HDI as reported by Wolff, Chong, and Auffhammer (2011) show that, although uncertainty exists for all the indicators, life expectancy is the least error‐prone among the three (see Appendix Figure B1).
3. It is built on data that are more clearly comparable across countries and times. Mortality data allow researchers to provide a much more consistent picture of development patterns at country levels, both geographically and historically.

Life expectancy at birth is the arithmetic average of ages at death, and like any other arithmetic average it is not influenced by the distribution of ages of death around that average. For example, two countries with the same life expectancy might have very different infant mortality figures, simply because in a country with low infant mortality, adults die earlier (perhaps as a result of genetics). The measure that we propose is a modification of life expectancy that also takes the distribution of ages at death around its average value into account.

If people experience the age‐specific survival rates in a life table, they will die at various ages. The arithmetic mean of their lifespans is life expectancy at birth. Our measure, called the Human Life Indicator (HLI), is the geometric average of those lifetimes:

$$HLI=\prod_{i=1}^{N}{\left(age_{i}+a_{i}\right)}^{d_{i}}$$

where $age_{i}$ is the age at the lower end of the age interval *i* in a life table, $a_{i}$ is the average number of years lived in the interval by those who die in the interval, $d_{i}$ is the fraction of deaths in age interval *i* among all deaths, and N is the number of age intervals in the life table.

The use of the geometric mean penalizes countries that have a relatively high variation in the length of lives. In particular, if two countries had the same life expectancy at birth, the country with the lower infant and child mortality rates would generally have the higher HLI. Infant and child mortality rates are highly correlated with education across countries and time periods, so using the HLI reduces the information lost by not explicitly including education. More generally, the HLI explicitly treats the reduction in the inequality in lifetimes as an additional contributor to the improvement in human development. If everyone in the population lived the same number of years (implying, in particular, a zero child mortality rate), the HLI and the life expectancy at birth would coincide.

Table 2 shows the correlations among the HLI, the HDI, gross national income per capita (GNI per capita), expected years of schooling (EYS), and mean years of schooling (MYS). Although the HLI is much simpler than the HDI, the correlation between the two is 0.93. The observation that the HLI, which is based on a single indicator, is so closely correlated with the HDI reflects the extent of redundancy in the HDI indicators. Table 2 also shows that the HLI is closely correlated to the two schooling variables. The effects of education are reflected in the HLI, even though the schooling variables are not explicitly included in it.

**Table 2:** Correlations between HLI and HDI and its components

	HLI	HDI	GNI/POP	EYS	MYS
HLI	1.00				
HDI	0.93	1.00			
GNI/POP	0.66	0.74	1.00		
EYS	0.84	0.92	0.63	1.00	
MYS	0.80	0.91	0.59	0.83	1.00

SOURCE: UNDP 2016b.

NOTES: Correlations between the Human Life Indicator (HLI), the Human Development Index (HDI), Gross National Income per capita (GNI/POP), Expected Years of Schooling (EYS), and Mean Years of Schooling (MYS). Based on all countries in the 2014 Human Development Report.

## The geography and history of the HLI across countries

**Figure 2:**
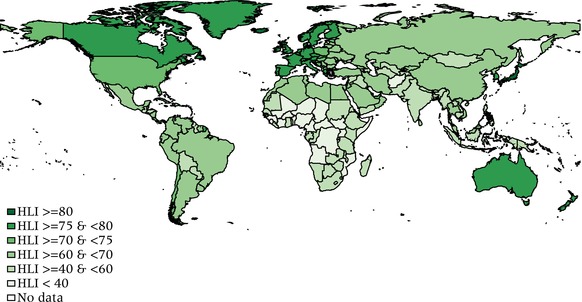
The HLI around the world SOURCE: UN 2017. NOTE: HLI = Human Life Indicator.

**Figure 3:**
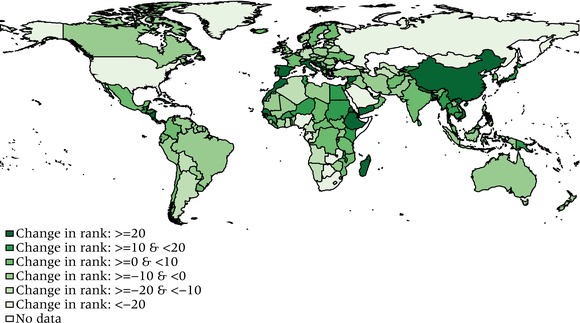
Changes in country rankings from a ranking based on the HDI to a ranking based on the HLI SOURCES: UNDP 2016b and authors’ calculations. NOTES: Positive numbers imply improvement in rankings. HDI = Human Development Index. HLI = Human Life Indicator.

Figure 2 shows the distribution of the HLI across countries based on 2010–15 UN life tables. In general, countries broadly maintain their development rankings when the measure switches from HDI to HLI (Figure 3). Nevertheless, the correlation between HLI and HDI is not perfect and for some countries the changes in the relative position can be substantial. This mainly happens for the (relatively) richer countries, where the high level of income represents the main driver of the good HDI performance. Consider for example Norway, which has been on the top of the HDI ranking for the last two decades. Its high GNI per capita is based, in part, on its extensive North Sea oil and gas deposits. When using the HLI, Norway slips to the ninth position, mainly because its very high levels of GNI per capita have not been translated into correspondingly longer life spans.

**Figure 4:**
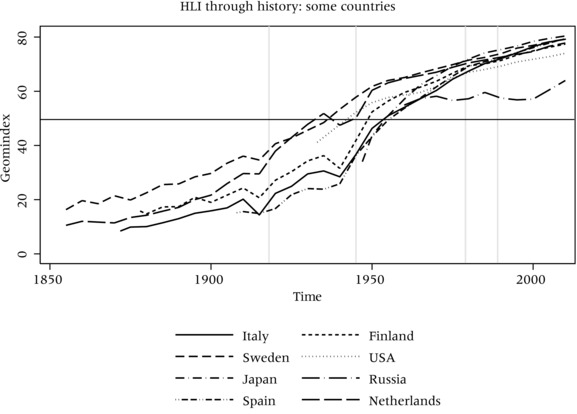
HLI for selected countries SOURCE: UC Berkeley, and Max Planck Institute for Demographic Research 2017. NOTE: HLI = Human Life Indicator.

**Figure B2:**
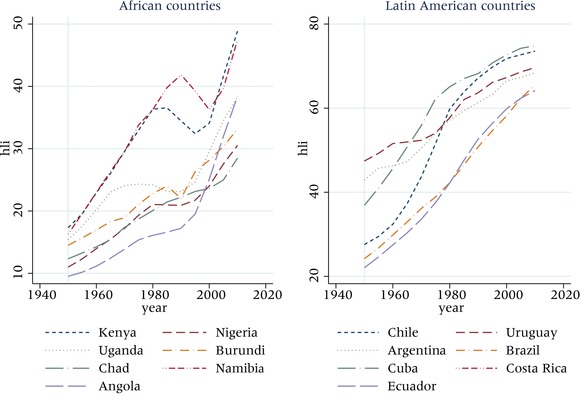
HLI in history, African and Latin American countries NOTES: Authors’ calculations based on UN 2017. HLI = Human Life Indicator.

**Figure B3:**
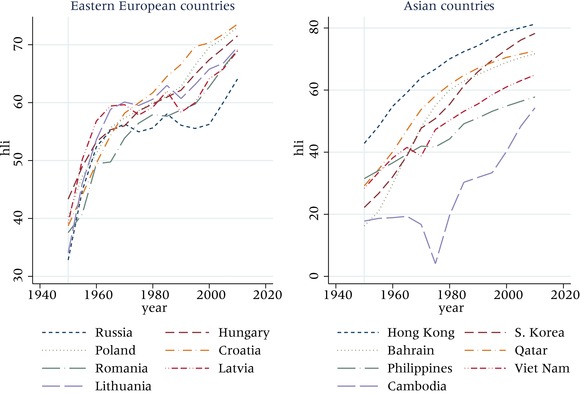
HLI in history, Eastern European and Asian countries NOTES: Authors’ calculations based on UN 2017. HLI = Human Life Indicator.

One crucial advantage of the HLI is its capacity to provide a measure of human development that can go farther back than 1990. Looking at the series of HLI by countries (Figure 4), the impact of relevant historical events, such as, for example, the World Wars for European countries, are evident. The HLI is thus particularly responsive to major macro‐level shocks, a characteristic that is of primary importance for the longitudinal dimension of an index. Regarding the patterns of development, the series show that, with the exception of the Northern European countries, the period between the end of World War II and the 1980s produced the most impressive worldwide increase in human development ever witnessed in human history. This growth slowed down earlier (during the 1960s) only for Russia, which has experienced a stall in the growth of the HLI ever since. Steady growth patterns in the HLI are noticeable in other countries around the world. For example, the HLIs in Brazil, Chile, and Ecuador have all been constantly growing since the 1950s (see Appendix Figures Figures B2 and B3). In Asia, the HLI reflects the impact of some dramatic historic events, such as the Vietnam War and the Pol‐Pot dictatorship in Cambodia.

## HLI in Indian states

HLIs can be calculated whenever life tables are available, including places where suitable data on education and economic output per capita are not available. The study of comparative human development in urban and rural areas is one example of this. Even if life expectancy at birth, the mean years of schooling, the expected years of schooling, and some measure of economic output per capita were available in each area, producing urban and rural HDIs would still be problematic. An area may have relatively high measured output because of mineral production, for example, but the people in the area could be quite poor because they receive little benefit from that production. Regional economic magnitudes that account for price differences can be calculated in a number of ways and the differences could have consequential effects on the HDI values.

In addition, most economic measures only count output that goes through markets. A higher proportion of output in rural areas might be in nonmarketed goods and services. Using flawed economic data could produce regional HDIs, which are misleading for policy purposes.

In this section, we provide an example of how HLIs can be used to quantify regional differences in human development across space and over time. Table 3 shows the HLIs for urban and rural areas of Indian states for which Sample Registration System (SRS) life tables are available for 2007–11, 2011–15, and for the country as a whole.

**Table 3:** HLI for Indian states in different years, urban and rural

	Urban			Rural		
State	2011–15	2007–11	Difference	2011–15	2007–11	Difference
Andhra Pradesh	62.91	59.04	3.86	54.86	49.32	5.53
Assam	62.33	56.83	5.51	47.67	43.99	3.68
Bihar	60.42	56.47	3.95	55.22	50.49	4.73
Chhattisgarh	57.15	n.d.	n.d.	50.64	n.d.	n.d.
Delhi	65.83	n.d.	n.d.	60.95	n.d.	n.d.
Gujarat	61.30	58.52	2.78	51.36	47.85	3.51
Haryana	59.05	55.19	3.86	53.50	49.00	4.51
Himachal Pradesh	66.95	63.70	3.25	58.10	56.06	2.04
India	61.93	58.27	3.66	52.59	48.51	4.08
Jammu & Kashmir	64.30	60.74	3.55	57.43	54.43	3.01
Jharkhand	62.12	n.d.	n.d.	53.31	n.d.	n.d.
Karnataka	63.17	60.27	2.90	56.04	51.13	4.91
Kerala	70.37	69.88	0.49	69.35	68.61	0.74
Madhya Pradesh	57.21	53.49	3.72	45.63	41.28	4.35
Maharashtra	67.66	63.83	3.83	61.28	57.66	3.62
Odisha	60.52	54.11	6.41	50.32	44.18	6.14
Punjab	65.53	60.77	4.77	59.62	54.61	5.01
Rajasthan	58.39	55.57	2.82	50.05	45.79	4.25
Tamil Nadu	65.33	61.95	3.38	60.84	57.51	3.33
Uttar Pradesh	53.15	49.66	3.48	46.04	42.88	3.16
Uttarakhand	63.57	n.d.	n.d.	59.70	n.d.	n.d.
West Bengal	64.59	61.68	2.91	58.81	56.56	2.26

SOURCE: Government of India 2018 and authors' computations.

NOTES: Human Life Indicator (HLI) in urban areas of states for which Sample Registration System (SRS) life tables are available, 2007–11 and 2011–15. “n.d.” indicates that no data were available to make the calculation of HLI. States are listed in order of their HLIs in 2011–15. Figures are rounded independently.

A full analysis of human development in India based on HLIs is beyond the scope of this article, so we highlight only a few observations from the table. In Table 3, the highest HLI values are for Kerala. Besides Kerala's high HLI values, the urban‐rural differences in HLIs are almost nonexistent. The state that had the greatest increases in both urban and rural HLIs between 2007–11 and 2011–15 was Odisha. Although Odisha had a relatively large urban‐rural difference in HLIs in 2007–11, the rapid increases in HLIs were somewhat faster in the urban area, increasing urban‐rural inequality in human development there. It is interesting to compare the experience of Odisha with that of Assam. For India as a whole, the urban‐rural gap in human development, as assessed using HLIs, diminished between 2007–11 and 2011–15. In rural India, the HLI increased by 4.08 in that period while in urban India it increased by 3.66. In Odisha, HLI increases in both the urban and rural areas were faster than those in India as a whole. In Assam, on the other hand, the urban HLI grew faster than the all‐India urban HLI, while the rural HLI grew more slowly than the all‐India rural HLI. Observations such as these can potentially be useful in assessing the sustainable development goal of reducing regional inequalities.

## Concluding remarks

No measure of the progress of human development is perfect. Different indices provide different perspectives. The HLI is simpler than the HDI and because of this does not presume contentious trade‐offs between the components of human development. Even though it is simpler, the correlation between the HLI and the HDI is rather high (0.93). The HLI does not explicitly include an economic component. In theory, this is a disadvantage, but in practice, it might not be. GNI per capita measured in purchasing power parity has been subject to large revisions each time new benchmark figures have been published. Moreover, there is a great deal of redundancy in the HDI components, so differences in economic conditions are still reflected in the HLI. The behavior of the HLI in time and space reflects the major economic and political events across the world and provides a credible picture of the evolution of human development in the last century.

Reducing inequality in human development is a sustainable development goal. The HLI can be useful in assessing progress toward that goal for two reasons. First, the HLI takes inequality of lifespans into account. Holding life expectancy at birth constant, areas with less inequality in lifespans have higher HLIs. Second, the HLI can also be used to study inequality in human development across regions of countries where it is difficult or impossible to obtain meaningful and comparable measures of economic conditions. The HDI and the HLI can both be used in policy analyses. One need not be an alternative to the other.

## Note

1 Purchasing power parity (PPP) is a method of adjusting the relative price levels so that equivalent amounts of currency would be able to buy an equivalent bundle of goods. For example, if, according to PPP calculations, 1 unit of currency in country A was the equivalent of 5 units of currency in country B, then a person with 1 unit of currency in country A and a person with 5 units of currency in country B should be able to buy the same amount of goods.

## APPENDIX A

### The Changing Definition of the HDI

**Table A1:** HDI and its components through the years

	LE index (x = LE)	Education index	Income index (y = income)	Income variable	HDI index mean
2005	$\frac{x-25}{85-25}$	$\frac{2}{3}\frac{alr}{100}+\frac{1}{3}\frac{ger}{100}$	$\frac{\ln(y)-\ln(100)}{\ln(40,000)-\ln(100)}$	GDP per capita	Arithmetic
2010	$\frac{x-20}{83.2-20}$	$\frac{1}{2}\left(\frac{mys}{15}+\frac{eys}{18}\right)$	$\frac{\ln(y)-\ln(163)}{\ln(108,211)-\ln(163)}$	GNI per capita	Geometric
2014	$\frac{x-20}{85-20}$	$\frac{1}{2}\left(\frac{mys}{15}+\frac{eys}{18}\right)$	$\frac{\ln(y)-\ln(100)}{\ln(75,000)-\ln(100)}$	GNI per capita	Geometric

NOTES: Formulas and parameters are taken from the Human Development Reports of the corresponding years. alr = Adult Literacy Rate; ger = Gross Education Rate; mys = Mean Years of Schooling; eys = Expected Years of Schooling. HDI = Human Development Index. LE = Life Expectancy.
